# The Day after Mass COVID-19 Vaccination: Higher Hypermetabolic Lymphadenopathy Detection on PET/CT and Impact on Oncologic Patients Management

**DOI:** 10.3390/cancers13174340

**Published:** 2021-08-27

**Authors:** Cristina Ferrari, Anna Giulia Nappi, Giulia Santo, Paolo Mammucci, Dino Rubini, Marco Tucci, Antonio Rosario Pisani

**Affiliations:** 1 Section of Nuclear Medicine, Interdisciplinary Department of Medicine, University of Bari Aldo Moro, Piazza Giulio Cesare 11, 70124 Bari, Italy; anna.giulia.nappi@gmail.com (A.G.N.); giuliasanto92@gmail.com (G.S.); paolo.mammucci@outlook.com (P.M.); rubini.dino@libero.it (D.R.); apisani71@libero.it (A.R.P.); 2 Division of Medical Oncology, Department of Biomedical Sciences and Human Oncology, University of Bari Aldo Moro, 70121 Bari, Italy; marco.tucci@uniba.it

**Keywords:** COVID-19, positron emission tomography, PET/CT, vaccination, axillary lymph nodes, oncologic imaging

## Abstract

**Simple Summary:**

The mass COVID-19 vaccination led to unexpected PET findings. Notably, axillary and interpectoral lymphadenopathies ipsilateral to the vaccine inoculation were observed, but could be wrongly interpreted, complicating cancer patients’ management. Our study aimed to assess the hypermetabolic lymphadenopathy detection rate on PET/CT and investigated factors that might help in lymphadenopathy interpretation. A significantly higher vaccine-related lymphadenopathy detection rate resulted in the vaccinated population, as well as in younger and vaccinated patients within 20 days before PET. SUVmax significantly changed during different time intervals, with the lowest values beyond 20 days. To minimize misdiagnosis, a detailed vaccination anamnesis must be recorded and should take into account the appropriate PET schedule, preferable to be performed 20 days after vaccine. Since this health emergency situation will probably continue in the near future, with the need for a strong vaccination campaign for the whole population, it is essential to keep in mind these considerations in order to better manage and take care of oncologic patients.

**Abstract:**

The widespread COVID-19 vaccination led to unexpected PET findings. Notably, axillary and interpectoral lymphadenopathies ipsilateral to the vaccine inoculation were observed. We aimed to assess the hypermetabolic lymphadenopathy (HLN) detection rate on PET/CT. Secondly, we investigated factors that might help in HLN differential diagnosis. A retrospective analysis on 1196 consecutive patients referred for a PET/CT was performed. All patients were asked about the date, type and site of vaccine injections. HLNs were recorded and categorized according to risk classes and SUVmax grades. A statistical analysis was performed to assess the correlation between HLN detection and different clinical/vaccine data. HLN detection rate was 15% and 27% in the No Vac- and vac-groups (*p* < 0.001), respectively. In the Vac-group, age (*p* < 0.001) and time interval from vaccine-to-PET (*p* = 0.010) were inversely correlated with HLN detection. Furthermore, SUVmax significantly changed during time intervals, with lower values beyond 20 days (*p* < 0.001). In the era of mass COVID-19 vaccination, a higher axillary and interpectoral lymphadenopathies detection ipsilateral to vaccine injection was observed. These PET findings can be wrongly interpreted, complicating cancer patients’ management. To minimize these pitfalls, a detailed vaccination anamnesis must be recorded and should take into account the appropriate PET schedule.

## 1. Introduction

On 27 December 2020, Italy started the mass vaccination program against the severe acute respiratory syndrome coronavirus 2 (Sars-CoV-2) infection and this date has already gone down in history as the “Italian V-day”, representing a day of rebirth for our country, as well as for the whole word.

First, the vaccination program involved healthcare workers in order to prevent hospital foci and preserve those who first faced the emergency; immediately after, the entire population received the first vaccine dose starting with the elderly, who are considered at higher risk of infection. Nowadays in Italy, more than half the population is vaccinated against COVID-19 with a booster vaccine dose.

As the vaccine is becoming widespread, new unexpected scenarios have arisen and these can affect the way we manage our medical routine. Namely, an increasing number of patients with vaccination against COVID-19 underwent positron emission tomography/computed tomography (PET/CT) for different indications and vaccination-related findings on PET images emerged. Starting from this evidence, a growing interest on imaging was developed, in order to prevent wrong interpretation that can impact clinical practice.

The most common reported vaccine-related finding on PET/CT was axillary, interpectoral, supraclavicular and lower cervical hypermetabolic lymphadenopathy (HLN) ipsilateral to the vaccine inoculation. Moreover, increased radiopharmaceutical-uptake in the deltoid muscle corresponding to the vaccine inoculation site was also frequently described. Less commonly, diffuse splenic [^18^F]FDG-uptake was reported [1]. These findings, related to [^18^F]FDG-avidity of infectious tissue and inflammation, were previously observed after antiH1N1 and antiHPV vaccination [2,3,4]. The misdiagnosis of equivocal lymphadenopathy can impact diagnostic accuracy of PET/CT in oncologic patients, affecting their management and complicating the diagnostic decision-making process about their healthcare.

The primary endpoint of this retrospective study was to assess the detection rate of HLNs in vaccinated patients who underwent PET/CT examination, compared with non-vaccinated patients. Second, we aimed to investigate the patients’ characteristics, vaccine- and PET-related factors that might help in correctly interpreting HLNs findings on the PET/CT exam.

## 2. Materials and Methods

### 2.1. Study Design and Population

This retrospective single-center study included all patients who underwent PET/CT scan for any clinical indication between 1 March 2021 and 30 June 2021. In this period, 1196 PET/CT exams were performed in our Nuclear Medicine department: 1025 [^18^F]Fluorodeoxyglucose (FDG) PET/CT, 112 [^18^F]Fluorocholine (FCH) PET/CT, 45 [^18^F]Flutemetamol PET/CT and 14 [^18^F]Fluciclovine PET/CT. All [^18^F]Flutemetamol PET/CT and 2/1025 [^18^F]FDG PET/CT exams were excluded because they were a cerebral acquisition only.

Clinical information, including age, sex, oncological status and current treatment, was recorded. In addition, data regarding vaccination were also collected: date of the first and, if applicable, booster vaccine dose, type of vaccine and site of injections, considering that most frail and cancer patients had early mRNA COVID-19 vaccination. Then, patients included in the analysis were classified in the Vac-group, including those who received at least the first vaccine dose before the PET exam, and the No Vac-group, which includes non-vaccinated patients and those who received the first vaccine dose after the PET scan. All patients gave their informed consent for the scientific use of medical data. Given the retrospective nature of the study, our Institutional Review Board does not require the Ethical Committee’s approval for review of the patients’ files.

### 2.2. PET/CT Acquisition

All PET/CT scans included in the study were performed according to our institute’s clinical scanning protocols. Acquisitions were performed on a Discovery 710 PET/CT scanner (GE, General Electrics, Milwaukee, WI, USA). The field of view and pixel size of the PET images reconstructed for fusion were 70 cm and 2.73 mm, respectively, with a matrix size of 256 × 256. The technical parameters used for CT imaging were: pitch 0.98, gantry rotation speed of 0.5 s/rot, 120 kVp, and modulated tube current of 140 mA. After 6 h of fasting, patients received an intravenous injection of 3 MBq/kg [^18^F]FDG or [^18^F]FCH and 370 MBq [^18^F]Fluciclovine. About 60 min after [^18^F]FDG/FCH administration or 4 min after [^18^F] Fluciclovine injection, CT images were obtained from the skull base to the midthigh. A 3D acquisition mode PET scan for the same longitudinal coverage, 2.5 min per bed position was performed. CT images were used for attenuation correction, anatomical information and images interpretation. Image analysis was carried out using a dedicate console (AW Server 4.7, General Electrics, Milwaukee, WI, USA).

### 2.3. Image Interpretation and Data Analysis

Two nuclear medicine physicians (C.F., A.R.P.) with at least 10 years of PET/CT reading experience reviewed all PET data sets. PET/CT findings were interpreted independently, taking into account the patients’ clinical data and vaccination status. In case of a disagreement, the readers discussed until an agreement was reached.

All cervical, axillary, supraclavicular and interpectoral HLNs ipsilateral to the vaccine injection were reported both in the Vac-group and in the No Vac-group, the latter one was used as control group.

In addition, a semiquantitative analysis was performed, drawing a semi-automated cubicle volume of interest (VOI) around HLN and measuring the corresponding maximum standardized uptake value normalized for body weight (SUVmax) within the VOI.

Based on radiopharmaceutical uptake, HLN was graded on a 3-point scale, as follows: grade 1 with mild uptake intensity (<2.2), grade 2 with moderate uptake intensity (2.2 ≤ SUVmax ≤ 4), grade 3 with high uptake intensity (SUVmax > 4).

Focusing on the Vac-group, HLNs were interpreted as benign vaccine-associated hypermetabolic lymph nodes (VAHL), malignant (MHL) or equivocal (EqHL) nodal involvement and grouped according to the relative risk class, taking into account the status (staging, restaging, follow-up) and extension (localized, metastatic) of the disease, by using clinical and instrumental data. Namely, HLN was interpreted as VAHL (a) if it was the only finding on PET in absence of other sites of abnormal radiopharmaceutical uptake, and/or (b) if not directly related to the primitive cancer site (e.g., contralateral breast/melanoma cancer) or to other possible causes of infection/inflammation (investigated during anamnesis), and/or (c) if a benign aspect on co-registered CT appeared. Conversely, HLN was considered as MHL in the presence of at least one of the following characteristics: (a) if systemic lymph node disease or other sites of abnormal radiopharmaceutical uptake were detected on PET, (b) if it was directly related to the primitive cancer site (e.g., ipsilateral breast/melanoma cancer or already detected on previous imaging), (c) if malignant aspect on co-registered CT was observed, and/or (d) in the presence of symptoms or signs of disease. Finally, EqHL was considered for any HLN not included in VAHL or MHL groups.

Furthermore, the time interval between vaccination and PET/CT was segmented in time periods, as suggested by the literature: 0–6 days, 7–19 days and equal or over 20 days [5].

### 2.4. Statistical Analysis

The statistical analysis was performed using IBM SPSS Statistic Version 28 (IBM Corporation, Armonk, NY, USA). Categorical variables are expressed as frequency distribution.

The HLN detection rate was estimated in the Vac-group and compared with the control group (No Vac-group). Then, it was correlated to clinical (age, sex, type, status and extension of disease, and ongoing treatment) and vaccine-related (type, number of doses, and number of days from vaccine to PET) variables.

Continuous variables were presented as mean ± standard deviation if normally distributed, or median (range) otherwise. The chi-square test and Fisher’s exact test were applied to compare proportion between the groups. Independent samples Mann-Whitney and Kruskal-Wallis test were used to compare continuous variables. For all comparison, a *p* value of <0.05 was considered to be statistically significant.

## 3. Results

### 3.1. Patients Characteristics

Out of 1196 PET/CT, beyond the aforementioned 47 brain-only scans, 337 additional exams were excluded from the analysis with a reason: 20 because they were performed on patients younger than 16 years of age and 317 for the lack of vaccination data. Among the remaining 812 patients, 707 were vaccinated and 105 received no dose. Of all vaccinated patients, 437/707 received at least the first dose before the PET scan (Vac-group; mean age 64 ± 14 years, range 21–88, female 205, male 232), while 270/707 were vaccinated after the PET scan and included in the No Vac-group.

**Figure 1:**
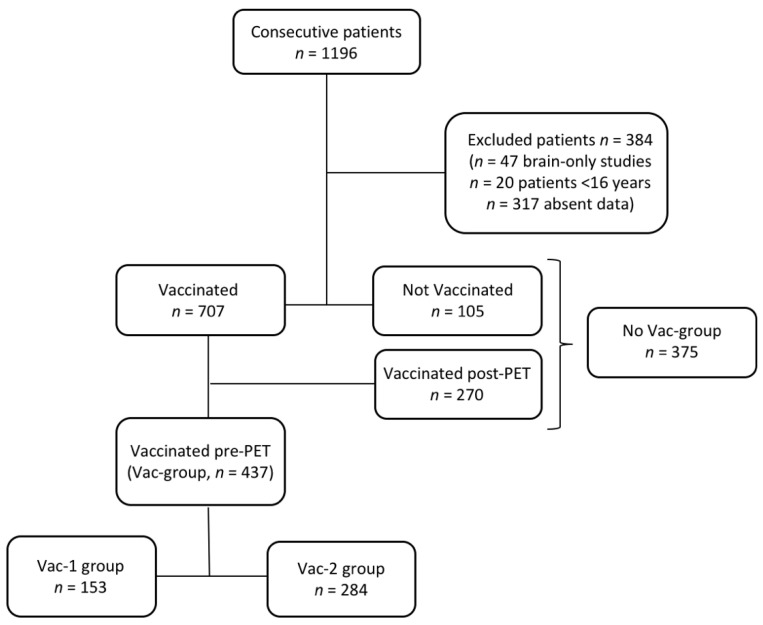
Patients flow chart.

In the Vac-group, 153/437 received only the first vaccine dose (Vac-1 group) and 284/437 received both the first dose and the booster vaccine dose (Vac-2 group) (Figure 1). The median time between vaccine doses and the PET/CT was 23 days (range 1–136 days). Demographic characteristics of the study population are summarized in Table 1.

### 3.2. Hypermetabolic Lymph Nodes Detection and Categorization

In the No Vac-group, HLNs were detected in 55/375 patients (15%), with a median SUVmax value of 5.3 (range 1.5–33.4), most of them graded as 3 according to the radiopharmaceutical uptake.

In the Vac-group, HLNs ipsilateral to vaccine injections were identified in 120/437 patients (27%), of which 41/120 (34%) were after the first vaccine dose and 79/120 were after the booster vaccine dose (66%), with a median SUVmax of 4.1 (range 1.4–24.5). No HLN was found in 317/437 (73%) vaccinated patients, of which 112/317 patients (35%) belonged to the Vac-1 group and 205/317 patients (65%) to the Vac-2 group.

The HLN detection rate was significantly higher in the Vac-group compared with the No Vac-group (*p* < 0.001), while no statistically significant difference in SUVmax value was found between these two groups (*p* = 0.141).

**Figure 2:**
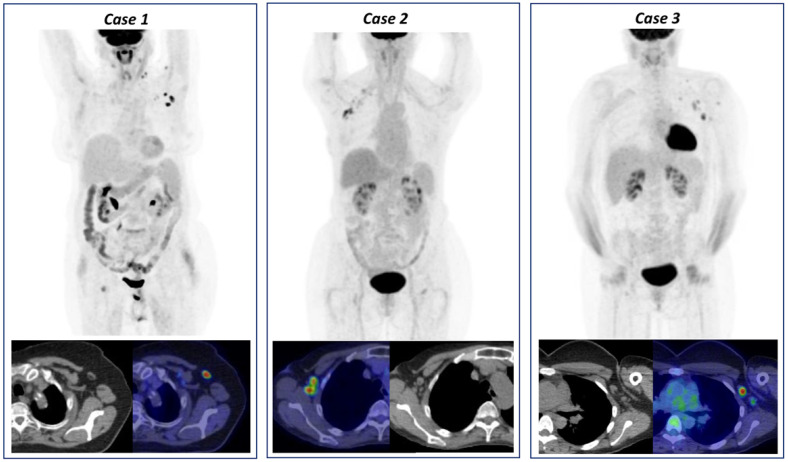
Three cases of vaccine-associated hypermetabolic lymphadenopathy (VAHL) detected on [^18^F]FDG PET/CT. Case 1: [^18^F]FDG PET/CT performed in a 67-year-old woman for left breast cancer follow-up and increased tumor markers. The images showed [^18^F]FDG-uptake in left supra- and retro-clavicular lymph nodes (maximum standardized uptake value, SUVmax 3.3) as well as homolateral axillary lymphadenopathy (SUVmax 7.0), classified as grade 3. Patient medical history reported the booster vaccine dose injection ipsilateral to PET findings 7 days before examination. Case 2: [^18^F]FDG PET/CT performed in a 42-year -old woman for melanoma of right dorsal region follow-up. The images showed [^18^F]FDG-uptake in right subclavicular and interpectoral lymph nodes (SUVmax 3.9), classified as grade 2. Patient medical history reported the booster vaccine dose injection ipsilateral to PET findings 16 days before examination. Case 3: [^18^F]FDG PET/CT performed in a 80-year-old man for follow-up of superior diaphragmatic non-Hodgkin lymphoma. The images showed [^18^F]FDG-uptake in left cervical, subclavicular and axillary lymph nodes (SUVmax 14.5), classified as grade 3. Patient medical history reported the booster vaccine dose injection ipsilateral to PET findings 2 days before examination. All these findings were VAHL, considering clinical history and the lymph nodes features on co-registered CT images.

The frequency of risk classes and SUVmax grade groups are reported in Table 2 and three representative clinical cases of VAHL are reported in Figure 2.

**Figure 3:**
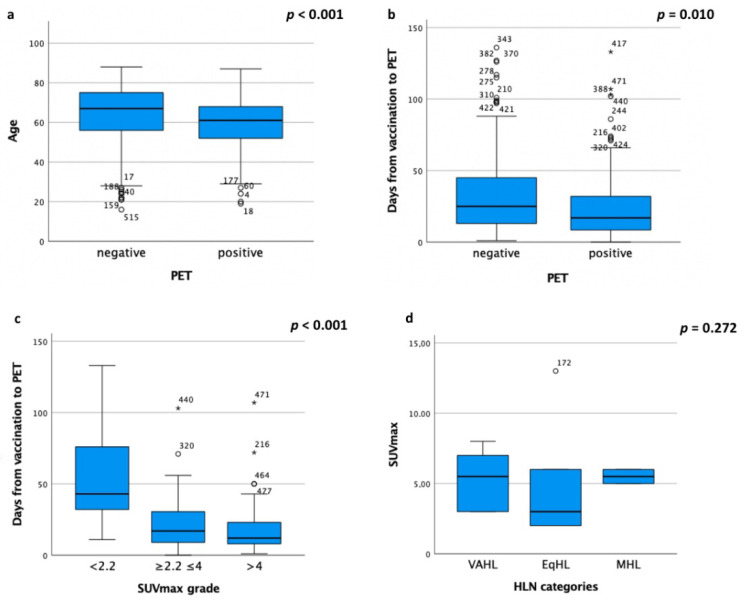
Box plots of (**a**) age and (**b**) days from vaccine-to-PET in PET findings, (**c**) days from vaccine-to-PET in SUVmax grade groups and (**d**) SUVmax value in HLN risk classes. ° Outlier outside the Upper Inner Fence; * Outlier outside the Upper Outer Fence. SUVmax: maximum standardized uptake value; HLN: hypermetabolic lymphadenopathy.

In the Vac-Group, age significantly correlated with HLN detection (*p* < 0.001), higher incidence in younger patients (Figure 3a). Notably, HLNs were identified in 68/182 (37%) patients under 65 years of age, while in 52/255 (20%) patients over 65 years of age.

A statistically significant inverse correlation was found between the number of days from vaccine to PET and HLN detection rate (*p* = 0.010) and this result was more evident beyond 20 days (no HLN:194/248, 78%, vs. HLN: 54/248, 22%) (Figure 3b).

**Figure 4:**
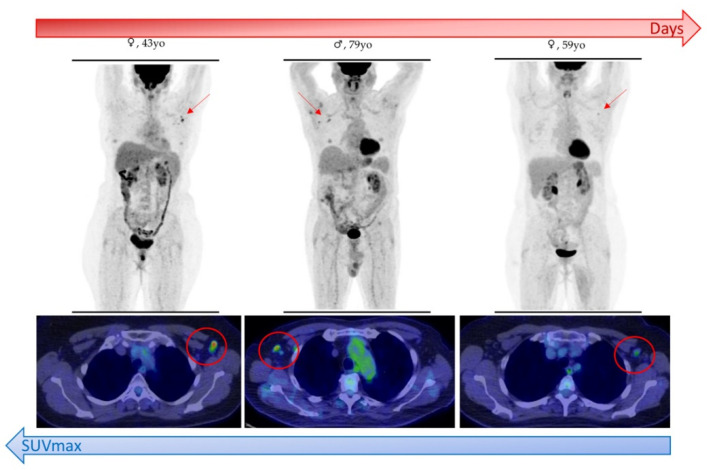
Schematic representation of inverse correlation between time interval from vaccination to PET/CT and maximum standardized uptake value (SUVmax) of vaccine-associated hypermetabolic lymphadenopathy (VAHL) detected in patients who underwent [^18^F]FDG PET/CT.

Furthermore, SUVmax significantly changed during different time intervals, with the lowest values beyond the 20-day interval from vaccine-to-PET (*p* < 0.001) (Figure 3c and Figure 4). Conversely, no statistical difference in SUVmax value was found in HLN risk class (*p* = 0.272) (Figure 3d).

None of the other variables resulted statistically significant, except for the PET radiopharmaceuticals (*p* = 0.002), as reported in Table 3.

Focusing on VAHL group, a statistical correlation between SUVmax grade and categorical/continuous variables was evaluated, as reported in Table 4. Even in this risk class, SUVmax significantly changed during different time intervals, with the lowest values beyond the 20-day interval from vaccine-to-PET (*p* = 0.004). No other variables significantly correlated with SUVmax grade in this subgroup.

## 4. Discussion

Since mass COVID-19 vaccination started, several evidences of PET/CT findings have emerged in literature, which mainly consisted in clinical case reports [6,7,8,9,10].

[^18^F]FDG-uptake in axillary, supraclavicular and cervical lymph nodes were commonly observed on PET images following COVID-19 vaccination. This finding was already observed after vaccination against influenza and papillomavirus, but the higher HLN incidences following COVID-19 vaccine can be justified by a more severe and longer reaction to mRNA biotechnology vaccines compared with traditional ones [11].

However, the major issue was to differentiate these benign lymphadenopathies from malignant ones, to avoid a misdiagnosis in frail patients with various types of disease, especially breast cancer, melanoma and lymphoma [11].

This retrospective study was concepted to face this need in a larger cohort of patients, identifying factors that can impact HLN detection rate and the PET schedule for oncologic patients. In the current study, among 437 vaccinated patients before the PET examination, 27% showed cervical, axillary, supraclavicular and interpectoral HLNs ipsilateral to vaccine injection, with a higher detection rate after the booster dose (booster dose: 29% vs. first dose: 27%), in younger patients (younger: 37% vs. older: 20%) and within 20 days from the vaccine (<20 days: 35% vs. ≥20 days: 22%). Considering different risk classes, the VAHL detection rate was 54% among all HLNs detected in the Vac-group.

A similar study was conducted by Cohen et al. on a 728 vaccinated patients cohort who underwent [^18^F]FDG PET/CT. The authors reported a higher incidence of HLNs in vaccinated patients (45.6%), especially in patients younger than 62–64 years of age, and an incidence of VAHL of 36.5% in the Vac-group, significantly higher after the second dose (45.8%) [5]. In accordance with our results, this higher HLN incidence in younger patients can be linked to a greater degree of immune activity stimulated by the mRNA vaccine [6,12].

Analogously to literature, our data suggested a significant correlation between the time interval from vaccine-to-PET and HLN detection, even if with a different trend. Indeed, Cohen et al. reported the highest HLN incidence in the 6–12 days interval after the first vaccination dose; while, in the Vac-2 group, the highest value of HLN incidence was observed immediately after the booster dose with a decrease in detection and SUVmax grade over time, reaching the lowest value beyond 20 days [5]. Similarly, our results demonstrated a reduction in HLN detection and SUVmax grade beyond 20 days from vaccination date, also confirmed by the VAHL sub-analysis. Therefore, no statistically significant difference between the Vac-1 and Vac-2 group was found both in the Vac-group analysis and in VAHL group sub-analysis. This different result can be explained by our recruitment period, in which most of patients had already received the second dose (Vac-1 group: 35% vs. Vac-2 group: 65%).

Unlike the literature [6], in our data, HLN detection rate was not influenced by oncological status, particularly by hematological disease and immunosuppressive/immunotherapy. In addition, differently from Adin et al., who observed a more common HLN detection rate after the Moderna vaccine (57%) than the Pfizer (15%) vaccine [9], the type of vaccination did not significantly impact on HLN detection; probably linked to the higher proportion (87%) of patients who received Pfizer vaccine in our sample.

In differentiating vaccine-related and malignant HLNs, SUVmax should not be considered as a discriminating parameter, since it did not significantly change in risk classes. However, to reduce false positive findings and correctly interpret PET images, a preliminary patient interview about the date of any prior vaccination should be executed to schedule PET examination appropriately. Performing the examination at least 20 days after the date of the vaccine can help in avoiding misdiagnosis due to the PET execution timing impact on SUVmax grade, as demonstrated in the current study. Our results were consistent with the recent National Comprehensive Cancer Network (NCCN) guidelines that recommended a delay of imaging by 4–6 weeks following COVID-19 vaccination [13,14], if this delay will not affect patient outcomes. If not possible, images must be interpreted more carefully taking into account both the oncological and vaccine anamnesis. However, the sufficient interval time to ensure the resolution of [^18^F]FDG-avid lymphadenopathy still needs to be investigated.

Moreover, the site of vaccine injection should be in the contralateral arm to the side of disease, especially in breast cancer, axillary lymphoma and malignancy of upper limbs patients [5].

**Figure 5:**
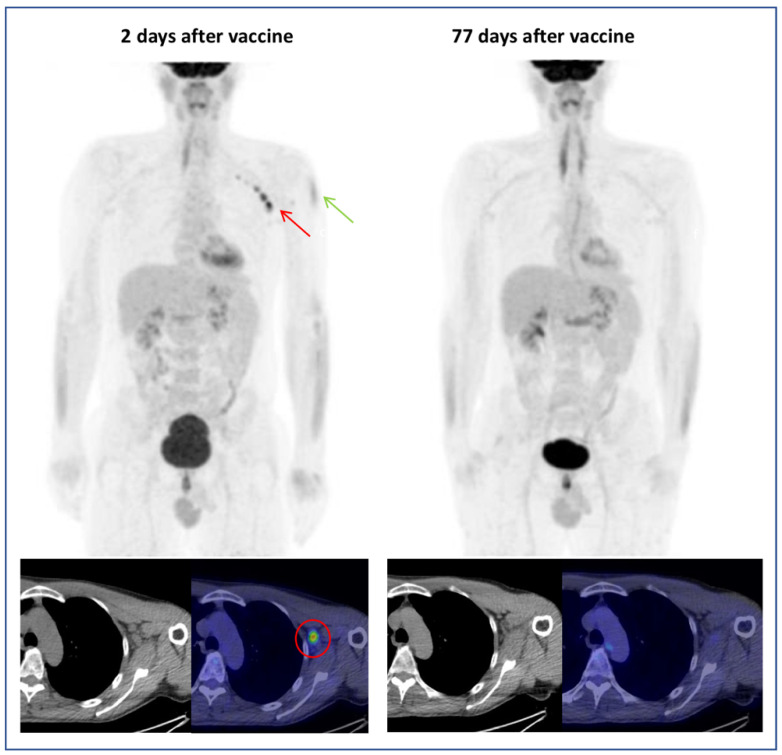
A 53-year-old man in follow-up for left supraclavicular melanoma (pT2aN0M0). [^18^F]FDG PET/CT exam performed 2 days after vaccination showed axillary (SUVmax 9.1) and interpectoral (SUVmax 3.5) hypermetabolic lymphadenopathies (HLNs) (red arrow) and [^18^F]FDG-uptake in left deltoid (injection site, green arrow). Being the HLNs ipsilateral to the site of the previous melanoma, a PET revaluation 77 days after vaccination was performed, showing the disappearance of FDG-avid lymphadenopathies in absence of therapy.

In case of EqHL, further assessment with ultrasound or short-distance PET examination can be suggested to clinicians (Figure 5). In addition, radiologists can contribute to an accurate HLNs interpretation, thanks to the evaluation of morphological features on CT co-registered images. Indeed, a moderate increase in lymph nodes size with a thickening of the cortex and fatty hilum suggests a benign lesion [11].

Our study presents some limitations. First of all, the lack of biopsy that histologically proved the HLN malignancy/benignity must be reported; analogously, a second scan and a consequent follow-up were not performed due to the short and recent period analyzed (121 days). Further studies, including these data, can be useful in order to confirm preliminary results. Secondly, most of the PET examinations were performed with [^18^F]FDG radiopharmaceutical; consequently, the statistically significant difference in the HLN detection rate among several PET radiopharmaceuticals should be considered with caution. Similarly, 87% of our patients were vaccinated with the Pfizer Covid-19 vaccine, so no reliable comparison could be performed with other mRNA vaccines and/or vaccine platform (e.g., viral vectors).

Surely, in the era of the COVID-19 pandemic, an accurate and detailed oncological and vaccination anamnesis must be collected in all cancer patients; thus, representing the first and simplest step to guide nuclear medicine physicians in correctly interpreting metabolic imaging, with the awareness of imaging patterns related to the immune response to vaccination.

Since these vaccine-related findings, future prospective and oncological clinical trials can consider [^18^F]FDG PET/CT as a potential theragnostic tool in assessing healthy lymphoid tissue for in vivo immune response quantification and in predicting immunotherapy outcome [15].

## 5. Conclusions

In the era of mass COVID-19 vaccination, a higher detection rate of vaccine-related PET findings is observed, particularly cervical, axillary, supraclavicular and interpectoral lymph nodes ipsilateral to vaccine injection. These findings can be wrongly interpreted, complicating the diagnostic decision-making process in cancer patients. To minimize these pitfalls, semi-quantitative analysis with SUVmax parameter did not represent a factor to help differential diagnosis. Consequently, a detailed anamnesis, including vaccination date, must be recorded and should be taken into account to appropriately guide the PET schedule, which is preferable to perform immediately before or 20 days after the vaccine. Since COVID-19 vaccination will probably be part of the healthcare routine in the near future, it is of utmost importance to keep in mind these considerations in order to better manage and take care of oncologic patients.

## Figures and Tables

**Table 1:** Demographic characteristics of the vaccinated study population.

Patients Characteristics	All-Vac Group (*n* = 437)	No Vac-Group (*n* = 375)
Mean age ± SD, years (range)	64 ± 14 (21–88)	61 ± 14.5 (16–87)
Female, *n* (%)	205 (47%)	178 (48%)
PET/CT indication, *n* (%)		
Hematological malignancy	107 (25%)	93 (25%)
Breast malignancy	53 (12%)	47 (13%)
Lung malignancy	76 (17%)	53 (14%)
Gastrointestinal malignancy	56 (13%)	45 (12%)
Gynecological malignancy	21 (5%)	15 (4%)
Genitourinary malignancy	61 (14%)	50 (13%)
Head and neck malignancy	23 (5%)	26 (7%)
Sarcoma	5 (1%)	4 (1%)
Melanoma	17 (4%)	17 (5%)
Inflammation/infection	10 (2%)	12 (3%)
Other malignancy	8 (2%)	13 (3%)
Extension of disease, *n* (%)		
Localized disease	162 (37%)	157 (42%)
Metastatic disease	275 (63%)	218 (58%)
Status of Disease, *n* (%)		
Diagnosis/staging	129 (30%)	96 (26%)
Interim/post-therapy	159 (36%)	146 (39%)
Follow-up	149 (34%)	133 (35%)
Treatment, *n* (%)		
No current treatment	306 (70%)	283 (75%)
Chemotherapy	55 (13%)	35 (9%)
Immunosuppressive/ Immunotherapy	27 (6%)	21 (6%)
Other	49 (11%)	36 (10%)
Vaccination data, *n* (%)		
Vac-1 group	153 (35%)	
Vac-2 group	284 (65%)	
Type of vaccination, *n* (%)		
BioNTech/Pfizer	380 (87%)	
Moderna	26 (6%)	
AstraZeneca	31 (7%)	
Median time vaccination-PET/CT, days	23	

**Table 2:** Frequency of risk classes and SUVmax grade groups in No Vac- and Vac-group.

Lymphadenopathy Characteristics	No Vac-Group (*n* = 375)	All Vac Group (*n* = 437)	*p*
HLN	55 (15%)	120 (27%)	<0.001
MHL		28 (23%)	
VAHL		65 (54%)	
EqHL		27 (23%)	
Median SUVmax	5.3 (1.5–33.4)	4.1 (1.4–24.5)	*p* = 0.141
Grading uptake			
Grade 1	6 (11%)	13 (11%)	
Grade 2	7 (13%)	45 (37%)	
Grade 3	42 (76%)	62 (52%)	

MHL: malignant hypermetabolic lymphadenopathy; VAHL: vaccine-associated hypermetabolic lymphadenopathy; EqHL: equivocal hypermetabolic lymphadenopathy; SUVmax: maximum standardized uptake value.

**Table 3:** Correlation between HLN detection rate and categorical variables in Vac-group.

Variables	Detection Rate	*p* Value
Sex, *n* (%)		<0.001
Female	46/230 (20%)	
Male	74/207 (36%)	
Type of disease, *n* (%)		0.599
Hematological disease	32/107 (30%)	
Solid tumor	83/316 (26%)	
Other	5/14 (36%)	
Type of treatment, *n* (%)		0.662
No treatment	85/306 (28%)	
Chemotherapy	12/55 (22%)	
Immunosuppressive/immunotherapy	7/27 (26%)	
Other	16/49 (33%)	
Type of vaccination, *n* (%)		0.141
BioNTech/Pfizer	103/380 (27%)	
Moderna	11/26 (42%)	
AstraZeneca/Vaxzevria	6/31 (19%)	
Number of vaccination dose pre-PET, *n* (%)		0.456
Vac-1	41/153 (27%)	
Vac-2	79/284 (28%)	
Number of days from vaccine to PET, *n* (%)		0.01
0–6	21/60 (35%)	
7–19	45/129 (35%)	
≥20	54/248 (22%)	
PET Radiopharmaceutical, *n* (%)		0.002
[^18^F]Fluorodeoxyglucose	117/389 (30%)	
[^18^F]Fluorocholine	3/45 (7%)	
[^18^F]Fluciclovine	0/3 (0%)	

**Table 4:** Correlation between SUVmax grade and categorical/continuous variables in VAHL group.

Variables	SUVmax Grade			*p* Value
	Grade 1 (<2.2)	Grade 2 (2.2–4)	Grade 3 (>4)	
Sex, *n* (%)				0.405
Female	5/43 (12%)	15/43 (35%)	23/43 (53%)	
Male	1/22 (5%)	11/22 (50%)	10/22 (45%)	
Type of malignancy, *n* (%)				0.109
Hematological disease	0/13 (0%)	3/13 (23%)	10/13 (77%)	
Solid tumor	6/50 (12%)	21/50 (42%)	23/50 (46%)	
Other	0/2 (0%)	2/2 (100%)	0/2 (0%)	
Type of treatment, *n* (%)				0.129
No treatment	3/46 (7%)	19/46 (41%)	24/46 (52%)	
Chemotherapy	0/5 (0%)	1/5 (20%)	4/5 (80%)	
Immunosuppressive/ immunotherapy	0/5 (0%)	2/5 (40%)	3/5 (60%)	
Other	3/9 (33%)	4/9 (45%)	2/9 (22%)	
Type of vaccination, *n* (%)				0.117
BioNTech/Pfizer	4/51 (8%)	22/51 (43%)	25/51 (47%)	
Moderna	0/9 (0%)	3/9 (33%)	6/9 (67%)	
AstraZeneca/Vaxzevria	2/5 (40%)	1/5 (20%)	2/5 (40%)	
Number of vaccination dose pre-PET, *n* (%)				0.334
Vac-1	1/24 (4%)	12/24 (50%)	11/24 (46%)	
Vac-2	5/41 (12%)	14/41 (34%)	22/41 (54%)	
Median time vaccine to PET, days (range)	37.5 (11–133)	19 (2-71)	12 (3-50)	0.004

## Data Availability

The data presented in this study are available on request from the corresponding author.
